# Feasibility, acceptability and efficacy of a web-based computer-tailored physical activity intervention for pregnant women - the *Fit4Two randomised controlled trial*

**DOI:** 10.1186/s12884-017-1277-9

**Published:** 2017-03-23

**Authors:** Melanie Hayman, Peter Reaburn, Matthew Browne, Corneel Vandelanotte, Stephanie Alley, Camille E. Short

**Affiliations:** 1 School of Medical and Applied Sciences, Central Queensland University, Rockhampton, QLD 4702 Australia; 2 School of Human, Health and Social Sciences, Central Queensland University, Bundaberg, QLD 4670 Australia; 3 School of Human, Health and Social Sciences, Central Queensland University, Rockhampton, QLD 4702 Australia; 4 NHMRC ECR Fellow. Freemasons Foundation Centre for Men’s Health. Faculty of Health Sciences, The University of Adelaide, Adelaide, SA 5000 Australia

**Keywords:** Physical activity, Pregnancy, Intervention, Behaviour change, Web-based, Internet, Online

## Abstract

**Background:**

Physical activity (PA) during pregnancy is associated with a variety of health benefits including a reduced risk of pregnancy related conditions such as pre-eclampsia and pregnancy-induced hypertension and leads to greater control over gestational weight gain. Despite these associated health benefits, very few pregnant women are sufficiently active. In an attempt to increase health outcomes, it is important to explore innovative ways to increase PA among pregnant women. Therefore, the aim of this study was to assess the feasibility, acceptability and efficacy of a four week web-based computer-tailored PA intervention among pregnant women.

**Methods:**

Seventy-seven participants were randomised into either: (1) an intervention group that received tailored PA advice and access to a resource library of articles relating to PA during pregnancy; or (2) a standard information group that only received access to the resources library. Objective moderate-to-vigorous physical activity (MVPA) was assessed at baseline and immediately post-intervention. Recruitment, attrition, intervention adherence, and website engagement were assessed. Questions on usability and satisfaction were administered post-intervention.

**Results:**

Feasibility was demonstrated through acceptable recruitment (8.5 participants recruited and randomised/month), and attrition (25%). Acceptability among intervention group participants was positive with high intervention adherence (96% of 4 modules completed). High website engagement (participants logged in 1.6 times/week although only required to log in once per week), usability (75/100), and satisfaction outcomes were reported in both groups. However, participants in the intervention group viewed significantly more pages on the website (*p* < 0.05), reported that the website felt more personally relevant *(p* < 0.05), and significantly increased their MVPA from baseline to post-intervention (mean difference = 35.87 min), compared to the control group (mean difference = 9.83 min) (*p < 0.05*), suggesting efficacy.

**Conclusions:**

The delivery of a computer-tailored web-based intervention designed to increase PA in pregnant women is feasible, well accepted and associated with increases in short-term MVPA. Findings suggest the use of computer-tailored information leads to greater website engagement, satisfaction and greater PA levels among pregnant women compared to a generic information only website.

**Trial registration:**

The trial was ‘retrospectively registered’ with the Australian New Zealand Clinical Trials Registry (ACTRN12614001105639) on 17^th^ October, 2014.

## Background

Participation in physical activity (PA) during pregnancy is associated with a variety of well-documented physical and physiological health benefits for both the mother and child [1]. These include greater control of gestational weight gain, decreased risk of pregnancy-related complications, such as preeclampsia and hypertension [1]. Despite these benefits, less than 35% of Australian pregnant women appear sufficiently active [2, 3] in accordance with exercise during pregnancy guidelines [4].

PA during pregnancy is linked to a variety of unique barriers experienced during pregnancy [5]. These include barriers such as increased fatigue and tiredness and physical discomfort, lack of time, motivation and/or social support, and neighbourhood or environmental barriers such as bad weather or poorly maintained walking paths [5]. To assist pregnant women overcome these barriers, PA interventions using a variety of strategies have been implemented, including counselling, exercise and educational interventions [6, 7]. Most of these PA interventions have traditionally included face-to-face behaviour change programs conducted in a primary care environment by exercise specialists and/or health professionals such as medical practitioners [6, 7]. However, such face-to-face behaviour change programs can be time consuming, expensive and limited in reach [8, 9]. Online interventions, especially those that employ computer-tailoring, present as an alternative intervention delivery mode to traditional face-to-face interventions; especially given that online interventions have been found to be cost effective, sustainable and wide reaching [10, 11].

Computer-tailoring automatically generates personalised feedback and advice based on participants’ responses to a series of online questions. While web-based, computer-tailored interventions have been shown to be effective at increasing PA among other populations, [11] their feasibility, acceptability and efficacy have not been examined among pregnant women. Thus, the purpose of this study was to test the feasibility, acceptability and efficacy of a computer-tailored web-based intervention (*Fit4Two*) designed to promote PA among pregnant women.

## Methods

This study was a randomised controlled, two-arm, four-week behaviour change trial with an immediate follow-up, conducted in Rockhampton, Queensland from October 2014 to June 2015. Ethics approval was obtained from Central Queensland University Human Research Ethics Committee (H14/02-031). The protocol was registered with the Australian New Zealand Clinical Trials Registry (ACTRN12614001105639). The reporting and conduct adheres to the Consolidating Standards of Reporting Clinical Trials (CONSORT) guidelines [12].

To be eligible, participants were required to be proficient in English, 18+ years, have a gestational age of 10–20 weeks (ensuring all participants completed the intervention in their second trimester for consistency), and considered healthy and free of any medical and/or obstetric contraindications to participate in PA. To ascertain contraindications, potential participants were required to complete a modified online version of both the Physical Activity Readiness Medical Examination for Pregnancy (PARmed-X for pregnancy) [13] and the Physical Activity Readiness Questionnaire [14]. If participants answered ‘yes’ to any of the screening questions, they were asked to seek medical approval to participate in the study.

Recruitment involved the dissemination of study information by medical practitioners, such as general practitioners and obstetricians, private and public hospital staff, and free and paid promotion of the study via social media. For all methods, a brief study overview was provided including information on the randomisation process. Following baseline assessment, participants were required to collect a *GeneActiv* original accelerometer (*ActiveInsights*, United Kingdom) for which content validity and test-retest reliability has previously been established in pregnant women, [15] from the study site. Participants were instructed to wear the accelerometer on their non-dominate wrist for seven days. Participants were also provided with an A4 instruction sheet including what should happen if the device stopped working, and general care instructions. Data was collected at 100Hz, with minimum wear time criteria consisting of >4 days, >10 waking hours/day and including two weekend days. Once objective baseline accelerometer data was successfully collected, participants were then automatically randomised to either the intervention or control group via the study website using a computer-generated block randomisations sequence (with block sizes of four) on a 1:1 ratio. All project team members were blinded to this process.

**Fig. 1:**
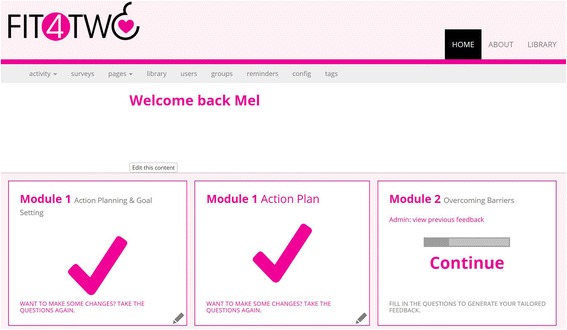
Fit4Two Website Homepage

**Fig. 2:**
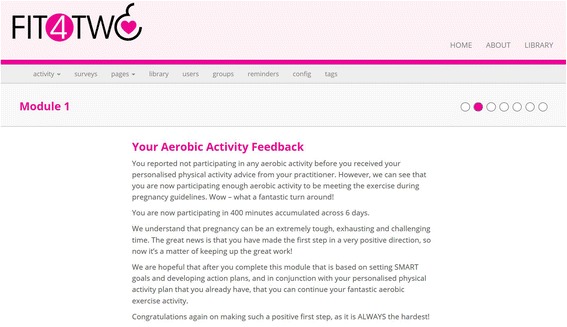
Example of Fit4Two aerobic feedback

Participants assigned to the intervention group were given full access to the *Fit4Two* website. The development of the website content was guided by the Social Cognitive Theory (SCT) given that self-efficacy is a central determinant of the SCT theory, and increasing self-efficacy is recognised as one of the most important determinants of physical activity among pregnant women [16]. The intervention consisted of four weekly modules based on Social Cognitive Theory (SCT) [17] including: (1) information on how to set SMART (specific, measurable, achievable, realistic and timely) goals and develop action plans; (2) overcoming barriers such as environment or lack of time and/or motivation; (3) developing social support networks including work colleagues, family and friends; and (4) PA reinforcement such as identifying the benefits associated with meeting the guidelines, all of which have been previously identified as significant constructs to increasing PA among pregnant women [6, 16]. In addition to the weekly SCT module, participants were also provided with access to a weekly action planning tool, which guided participants in setting a detailed plan for their PA including when they would exercise, who they might exercise with, where the exercise would take place, and what barrier/s might come up, and how they would overcome them over the coming week. Please refer to Table 1 for the operationalisation of the SCT constructs relevant to the Fit4Two study. Figures 1 and 2 show the look and feel of the intervention website.

**Table 1:** Operationalisation of the SCT Constructs relevant to this study

Module 1		
Strategy	SCT construct	Tailoring variables
Advice for meeting the PA guidelines	Self-efficacy	PA status PA level prior to pregnancy
Information about the beneficial outcomes of PA, exercise during pregnancy guidelines, resistance-based exercise guidelines and stretching	Outcome expectations	Outcome expectancies (outcomes valued by individual)
Advice and information on BMI classifications	Self-efficacy Behavioural capability Outcome expectations	Behavioural capability Outcome expectancies (outcomes valued by individual)
Advice on exercise intensity	Self-efficacy	PA status Current PA level
Advice on exercising safely	Self-efficacy Behavioural capability Outcome expectations	Behavioural capability Outcome expectancies (outcomes valued by individual)
Action planning	Self-efficacy Self-control and performance	PA status PA preference
Module 2		
Feedback on PA performance	Self-efficacy Self-control and performance Reinforcement	PA status PA progress since M1
Advice on overcoming possible barriers to exercise during pregnancy	Environment Self-efficacy Self-control and performance	Self-discipline Time Health/illness Tired and exhausted Motivation Social Support Knowledge Environment
Action planning	Self-efficacy Self-control and performance	Goal setting behaviour after M1 PA status
Module 3		
Strategy	SCT construct	Tailoring variables
Feedback on PA performance	Self-efficacy Self-control and performance Reinforcement	PA status PA progress since M1 and M2
Advice and tips on increasing social support to exercise during pregnancy	Environment Self-efficacy Self-control and performance	Partner Family and Friends Work colleagues
Action planning	Self-efficacy Self-control and performance	Goal setting behaviour after M1 and M2 PA status
Module 4		
Feedback on PA performance	Self-efficacy Self-control and performance Reinforcement	PA status PA progress since M1, M2 and M3
Reminder on Exercise during pregnancy guidelines and recommendations	Self-efficacy Self-control and performance Reinforcement	PA status PA progress since M1, M2 and M3
Action planning	Self-efficacy Self-control and performance	Goal setting behaviour after M1, M2 and M3 PA status

Upon commencing the intervention, participants were asked a series of questions based on their PA behaviours [18] over the previous seven days, and the weekly SCT constructs (goal setting and action planning, social support, perceived barriers and PA reinforcement). Participants were then provided with immediate computer-tailored feedback messages based on their responses to the PA behaviour and SCT construct questions. For example, if a participant answered that her friends and work colleagues were the ones that provided her with social support to be physically active during her pregnancy, the tailored feedback addressed this. The website also included a resources library providing up-to-date, evidence-based advice on current exercise during pregnancy guidelines, aerobic and resistance-based exercise recommendations, safety considerations to exercise during pregnancy, and general advice for active living during pregnancy. Participants assigned to the minimum intervention control group only had access to the resources library for the four-week intervention phase. Upon completion of the intervention phase, participants in the control group were then given unlimited access to the website.

Feasibility was assessed using recruitment, and attrition data. Acceptability was measured through intervention adherence, website engagement, website usability and satisfaction items relating to website quality, relevance and usefulness. Specifically, intervention adherence was measured by number of modules and goal setting/action planning tools that were completed. Website engagement totalled the number of site sessions (log-ins), page views and time spent (minutes) on the website from baseline to four week follow-up using Google Analytics [19]. Website usability was measured using the widely accepted and reliable 10-item System Usability Scale (SUS) [20]. All items were assessed on a five-point Likert scale with response items ranging from ‘strongly agree’ to ‘strongly disagree’. Reverse coding was used to adjust scores for negatively-framed items before participants’ response scores were added together for all 10 items and then multiplied by 2.5. This allowed for the original scores ranging from 0 to 40 to be converted to a score of 1–100. Based on previous research, a score above 68 on the SUS scale indicates ‘above average usability’ [20]. Finally, participant’s perceptions of the website in terms of how much they would like to continue to use it, and how credible, interesting, easy to understand, and personally relevant they found the website were assessed using five items from previous tailoring studies on a five-point Likert scale (1 – strongly disagree – 5 strongly agree) [21, 22]. For example, participants were asked to rate their level of agreement that *‘the content was personally relevant to me’* and ‘*I would like to continue using the Fit4Two website throughout the remainder of my pregnancy’*. Two open-ended questions were also used to ask participants; (1) what they liked and/or disliked about the website and; (2) how they believe the website could be improved.

Efficacy was determined based on a 2×2 group X time interaction model describing differences in moderate-vigorous physical activity (MVPA), controlling for baseline differences. The *GeneActiv* accelerometer data was used to assess MVPA objectively at baseline (seven days prior to randomisation) and follow-up (seven days after completing the *Fit4Two* study). At the end of each collection period, the raw accelerometer output data was uploaded into the *GeneActiv* post-processing software (GeneActiv, version 2.2, Activinsights Ltd) and converted into 60 s epoch files. The epoch files were then processed using the GGIR script in the R environment (http://cran.r-project.org) [23] to produce a series of standardised accelerometery outcome variables. Finally, validated acceleration magnitude cut points were used to classify activity into light, moderate and vigorous intensities [24].

Chi-square and analyses of variance were performed using *SPSS Version 20* (IBM Corp, NY) to determine between group differences for all measures of participants who completed the study, with the exception of MVPA [25]. The distribution of MVPA residuals violated normal assumptions due to excessive zeroes (33% of all observations) and over-dispersion (positive skew). Whilst specialised generalised distributions are available to handle this, these are not implemented for repeated measures/linear mixed effects models required to handle the repeated measures. Therefore, for the purpose of hypothesis testing, the over-dispersion was dealt with by transforming the response via log(MVPA + 1). The zero-inflation was handled with recourse to the semi-parametric bootstrap (5000 replicates) for robust calculation of confidence intervals. The model included main effects for time (pre, post) and group (intervention, control), with the hypothesis addressed by a group X time interaction term. Statistical significance was set at *P* < 0.05 (two-tailed, corresponding to 0.025 one-tailed).

## Results

**Fig. 3:**
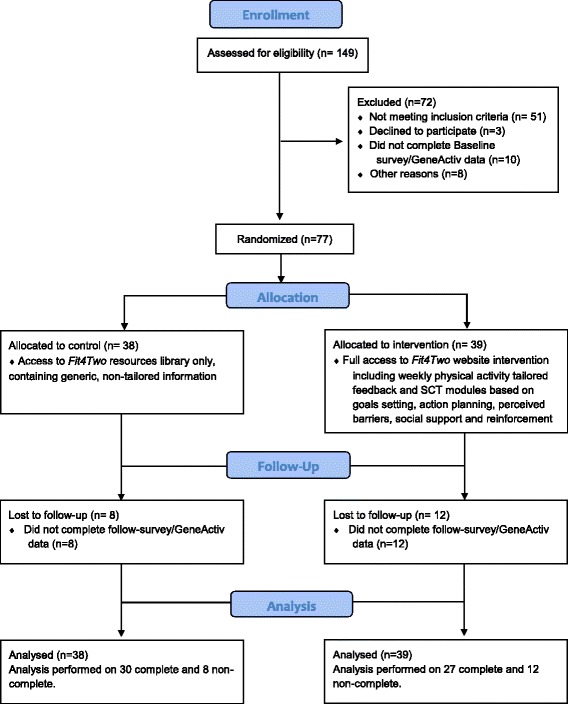
CONSORT Flow Chart of Participants through the Fit4Two Study

A total of 425 ‘guests’ visited the *Fit4Two* website over the nine-month recruitment period, with 149 women expressing interest in the study and screened for eligibility. Seventy-seven eligible participants provided informed consent by agreeing to the study terms and conditions listed on the *Fit4Two* website, and by choosing to continue in the study to complete the baseline survey, and wear an accelerometer in accordance with the study’s adherence guidelines. These participants were then randomised to either the control group (*n* = 38) or intervention group (*n* = 39). The main reasons for ineligibility were geographical location or gestational age being greater than 20 weeks. Of these 77 randomised participants, 18% were recruited from marketing materials displayed in doctor/hospital/obstetrician clinics, 9% were recruited via family and/or friends, and 59% via media outlets (*Facebook* 47%, community news 3%, other 9%). There was no significant difference in attrition rates between groups. Twenty participants dropped out of the study including 8 from the control group (80% retention) and 12 from the intervention group (70% retention). The flow of participants through the study is displayed in Fig. 3.

Baseline characteristics for all participants are shown in Table 2. There were no significant demographic differences between groups. Compared to available national data, participants were found to be representative of the target population [26] for age (mean 29 years), BMI (mean 26 kg/m^2^), combined household income (62% earned less than $100,000 per year), and highest level of education (51% completed a university degree).

**Table 2:** Demographic characteristics of Fit4Two participants

Variable	Intervention		Control		Total	
	(*n* = 39)		(*n* = 38)		(*n* = 77)	
	n	%	n	%	n	%
Married, de facto	34	87	34	89	68	88
Completed university	19	49	20	53	39	51
Combined Household Income ($100,000-$150,000 per year)	12	31	17	45	29	38
Full-time employed	17	44	21	55	38	49
Born in Australia	31	79	37	97	68	88
One other child at home	21	54	15	39	37	48
Have been pregnant before	27	69	23	60	50	65
Private Health Care	28	72	26	68	54	70

Feasibility, acceptability and efficacy outcomes are shown in Table 3. Of the participants who completed the study and were allocated to the intervention group (*n* = 27), 96% completed all four of the weekly SCT-based modules. Adherence to the Goal Setting/Action Planning tool was also positive (72%), with all participants completing Action Plans 1 and 2, 59% (*n* = 16) completing Action Plan 3 and 30% (*n* = 8) completing Action Plan 4.

**Table 3:** Feasibility, acceptability and efficacy of Fit4Two participants

Measure	Intervention (*n* = 27)		Control (*n* = 30)	
	N	%	N	%
Acceptability measures				
Adherence				
Module 1	27	100	N/A	
Module 2	27	100	N/A	
Module 3	26	96	N/A	
Module 4	27	100	N/A	
Action Plan 1	27	100	N/A	
Action Plan 2	27	100	N/A	
Action Plan 3	16	60	N/A	
Action Plan 4	8	30	N/A	
Website engagement				
Website session (log ins)	7		6	
Website page views	144*		110	
Average time spent on website (mins/session)	12		11	
Usability				
SUS Scale (score out of 100)	75		75	
Satisfaction data				
Found the website credible	19	71%	23	77%
Found the website interesting	21	78%	21	70%
Content easy to understand	23	85%	23	80%
Content was personally relevant to me	19*	71%	18	60%
Would like to continue using the website throughout pregnancy	21	78%	22	74%
Efficacy measures (objective pre-post MVPA mean change scores)				
Objective	+36 min per week*		+9 min per week	

*p < 0.05

Website engagement was highest in the intervention group, with participants in this group recording more sessions (log-ins), spending longer on the website and viewing significantly more pages (*p* < 0.05), than those in the control group. All participants scored the usability of the website ‘above average’ with a mean score of 75/100 [20]. Participant satisfaction was positive with the majority of participants either ‘strongly agreeing’ or ‘agreeing’ that website as credible, the content was easy to understand, and that they would like to have continued to use the website throughout the remainder of their pregnancy. No significant between-group differences were observed for any measure of acceptability with the exception of perceived content relevance, where participants in the intervention group felt the website content was significantly more relevant to them personally than those in the control group (*p* = 0.024). Whilst many participants said ‘they would not change a thing’, some participants suggested that the inclusion of actual exercise programs would have benefited the program.

Table 3 shows that at follow-up, participants in the intervention group were found to have increased their MVPA (mean increase 36 min/week) more than those in the control group (mean increase 9 min/week). This group x time interaction was significant when MVPA was modelled on the log scale using bootstrapped confidence intervals, *β*_*group X time*_ = 1.31, 95% *CI *[0.175, 2.484], *p* = 0.0126. There were no adverse events, or unintended harm reported from participants in this study.

## Discussion

The present study is the first study to explore the feasibility, acceptability and efficacy of a web-based computer-tailored intervention aimed at increasing PA among pregnant women. The feasibility of the *Fit4Two* program was demonstrated in terms of successful recruitment and attrition throughout the screening process with no participants dropping out between eligibility and randomisation. Acceptability was also established, with the majority of participants rating the website positively and reporting above average usability scores. Almost all participants allocated to the intervention group engaged with the tailored modules and action plans as intended. Finally, there was evidence in favour of the *Fit4Two* program in terms of efficacy, with those allocated to the intervention group showing a significant increase in MVPA compared to the control group.

The feasibility outcomes in this study are favourable compared to other studies [27–29]. For example, the present study recruited and randomised an average of 9 participants per month from targeted marketing in a regional Australian town with a population of approximately 65,000 people [30]. In comparison, a recent study examining the feasibility and efficacy of a PA mobile health intervention among Australian pregnant women from a metropolitan area of nearly 600,000 people, [27] recruited and randomised 5.6 participants per month [27]. Almost half (47%) of the participants within this study were recruited via the social media platform, *Facebook*. In this study, paid advertisements that included the Fit4Two study logo, and targeted central Queensland pregnant women wanting to know more about physical activity during pregnancy were used, and found to be a very effective recruitment tool. This is not surprising however given the fact that pregnant women are already reported to be utilising the web to source, and seek out, pregnancy related information. Specifically, the emergence of information technology sees over 93% of pregnant women using the internet to source additional information about their pregnancy with 83% using eHealth information to assist in decision-making during their pregnancy [31, 32].

The attrition rate in the present study (26%) is considered acceptable for PA web-based interventions undertaken in non-pregnant populations, [33] and also fell well within the lower range of attrition rates reported for PA interventions among pregnant women (0–43%) [6]. Web-based interventions are commonly associated with larger attrition among participants (up to 80%), more so among participants allocated to intervention/treatment groups compared to those of a control group [30]. In the present study, more participant drop-outs were observed in the intervention group (30%) compared to the control group (21%). This finding is not uncommon in interventions, [34] given the greater burden placed on intervention participants [34].

Participant acceptability in terms of adherence, website engagement, usability and process evaluation data in our study was very high compared to other web-based studies among pregnant women [27–29]. All but one participant completed all four of the weekly SCT modules. A total of 78/108 (72%) goal setting/action planning tasks were also completed. This finding is positive when compared to previous studies that have used goal setting and action planning tools in both pregnant women [27] and other populations [34]. However, in line with other studies, the use of the action plan tool did decline overtime. There may be a number of explanations for this observation. For example, some participants may not have felt that they needed to continue to set goals or develop action plans after the first two weeks of the intervention because they were successfully increasing their PA. Alternatively, participants may have felt that setting goals and developing action plans on a weekly basis was too demanding. Irrespective of the reasoning behind this decline, it is important that participants are aware of the positive relationship between setting goals, developing action plans, and overcoming barriers to PA [6, 35].

Participants in this study logged in an average of 1.63 times per week. This is a positive finding given that participants in the intervention group were only required to log-in once a week, and participants in the control were only required to log in once throughout the entire intervention period. This figure is higher than studies previously undertaken in pregnant women [27]. For example, in a recent web-based intervention aimed at increasing PA among pregnant women, 64% (29/45) of participants were reported as having ‘no engagement’ or ‘low engagement’ with the website tools, characterised by number of log-ins and interaction with the website content [27]. Possible explanations for the differing findings may be that the *Fit4Two* website included activities designed to help bring about positive behavioural changes, or that participants received computer-tailored feedback that was immediate, personalised, and based on their reported PA behaviours and perceived barriers to PA [27]. Alternatively, participants found the resources library section of the website useful and visited it frequently.

Participant satisfaction data was positive. However, participants in the intervention group reported that the content of the website was significantly more relevant to them than the participants in the control group. Interventions utilising computer-tailored messaging have previously been shown to be more effective at increasing PA (in other populations) than generic, non-tailored messaging, and this is believed to be owing to increased personal relevance [10, 11, 33]. Thus, the use of computer-tailoring in PA interventions among pregnant women is recommended to increase participant acceptability and efficacy. However, participants reported that the provision of specific exercise programs would have further enhanced the Fit4Two website. This request is supported by the literature in that some women still perceive exercise during pregnancy as somewhat ‘unsafe’ and are ‘unsure’ as to what exercise they can and cannot do [35, 36]. The reason for not providing exercise programs as a part of the *Fit4Two* program was because the research team did not believe the provision of generic programs would be safe for all participants, thus not taking into consideration their health status, physiological differences and possible contraindications to physical activity. However, future interventions might look to involve an accredited exercise physiologist (AEP) or other appropriately skilled professional to provide this service. Alternatively, the intervention could incorporate a referral tool that helps to link pregnant women with local AEPs within their area.

Finally, the efficacy of the *Fit4Tw*o project was confirmed with the intervention group significantly increasing their MVPA in comparison to the control group. This increase in MVPA is most likely a result of the personalised and relevant computer-tailored feedback that included appropriate behaviour change techniques (BCTs), identified by the use of the SCT theoretical framework [12, 29]. The benefit of using theories such as the SCT is that they help identify key determinants that assist in the selection of appropriate behaviour change techniques to overcoming the determinants of PA in specific cohorts, thus increasing the personal relevance of the intervention [16]. In the present study, participants in the intervention group were taught how to set goals and develop action plans, overcome perceived barriers to PA, and how to develop social support networks. Each of these BCTs has previously been identified as major facilitators to increasing PA among pregnant women [6, 7, 16].

Taken together, the findings in this study suggest that the *Fit4Two* program is feasible, acceptable and efficacious. The use of the SCT theoretical framework used to guide the development and design of the program, and the incorporation of computer-tailored messaging are two recognisable strengths of this study. Additional strengths of this study include the use of objectively measured MVPA and website engagement. A limitation to this study was that the only follow-up occurred immediately after the completion of the intervention. It is recommended that future studies also consider an additional follow-up time point to access the long-term efficacy of the intervention. Based on the positive findings of the present study, a larger trial of *Fit4Two* undertaken across a nationally representable sample is warranted.

## Conclusions

The positive feasibility, acceptability and efficacy findings associated with the present study suggests that the delivery of a web-based computer-tailored intervention for pregnant women presents as a promising approach to increasing PA among pregnant women. A larger study undertaken across a nationally representable sample is now recommended.

## Abbreviations

AEP: Accredited exercise physiologist
BCT: Behaviour change theory
MVPA: Moderate-to-vigorous physical activity
PA: Physical activity
SCT: Social cognitive theory
